# Moving through a changing world: Single cell migration in 2D vs. 3D

**DOI:** 10.3389/fcell.2022.1080995

**Published:** 2022-12-20

**Authors:** Anna Pawluchin, Milos Galic

**Affiliations:** ^1^ Institute of Medical Physics and Biophysics, Medical Faculty, University of Münster, Münster, Germany; ^2^ Cells in Motion Interfaculty Centre, University of Münster, Münster, Germany; ^3^ CIM-IMRPS Graduate Program, Münster, Germany

**Keywords:** cell migration, cytoskeletal forces, extracellular matrix, self-organization, membrane curvature

## Abstract

Migration of single adherent cells is frequently observed in the developing and adult organism and has been the subject of many studies. Yet, while elegant work has elucidated molecular and mechanical cues affecting motion dynamics on a flat surface, it remains less clear how cells migrate in a 3D setting. In this review, we explore the changing parameters encountered by cells navigating through a 3D microenvironment compared to cells crawling on top of a 2D surface, and how these differences alter subcellular structures required for propulsion. We further discuss how such changes at the micro-scale impact motion pattern at the macro-scale.

## 1 Introduction

How cell motion is accomplished in a 3D microenvironment, and to which degree observations acquired in a planar system can be translated into such a heterogenous environment, is object of intense ongoing research and the topic of this review. This essay is divided into two sections: a general introduction into the field, and a detailed analysis of how environmental factors affect a specific signaling circuit at the leading edge (LE) of mesenchymal cells. We begin the first section with a brief survey on the molecular mechanisms driving cell motion, and their emerging motion patterns in a planar system. Next, we review published work describing how changes in material properties impact force transmission and motion patterns. Following, we summarize which parameters differ when cells migrate on top of a 2D surface compared to cells embedded in a 3D environment, and how mechanical properties determine what mode of motion is employed by cells. The second section is focused on a self-organizing signaling circuit present at the LE of cells migrating in a mesenchymal mode. Considering the relevance of membrane geometry for the assembly of this signaling circuit, we begin the second part with an introduction on curvature-dependent protein activation, before exploring how mechanical properties may affect this signaling circuit and in consequence change the motion pattern employed by the cell. Along the whole review, we try to identify open questions and current limitations in the field.

## 2 Single cell migration in 2D vs. 3D

Migration of single cells on a 2D surface was first described more than half a century ago by pioneers of this field (Abercrombie, 1980; Ingram, 1969; Weiss, 1961; among others). Since then, many of the underlying mechanisms responsible for cell propulsion have been unveiled. We begin by summarizing the general properties of mesenchymal cell motion. Similarities and differences to other migration modes will be discussed later in the review.

### 2.1 Force transmission at the leading edge of migrating mesenchymal cells in 2D

The prerequisite of any form of directed force transmission is an initial symmetry breaking step, which determines the future front and rear end of the cell (Lauffenburger and Horwitz, 1996). Symmetry breaking is fueled by various internal and external signaling cues (Drubin and Nelson, 1996). One of the main sensors for polarized chemotactic signaling cues are G protein-coupled receptors (Rodríguez-Frade et al., 1999). At the molecular level, receptor activation converges into the polarization of commonly used secondary messengers. For instance, the LE displays increased phosphotyrosine kinase activity and elevated levels of PI (3,4,5) P_3_ and DAG, while cytosolic Ca^2+^ levels are lowered (Tsai et al., 2014). This polarized distribution of secondary messengers is associated with a non-isotropic distribution of small Rho GTPase activity that either promotes actin polymerization (e.g., Rac, Cdc42) or actomyosin contractility (e.g., RhoA). Importantly, as small GTPases transition between an active and inactive state, such a polarized activity does not necessarily rely on a redistribution of the GTPase itself, but may also be coordinated through relocation of actuators (i.e., GTPase exchange factors, GEFs) and inhibitors (i.e., Guanine nucleotide exchange factors, GEFs) of the respective Rho GTPase. Analysis of Rho GTPase activation presents an intriguing spatio-temporal pattern, with high levels of GTP-Rac and GTP-Cdc42 at protruding sites, while contractile regions show increased GTP-RhoA levels (Kraynov et al., 2000; Nalbant et al., 2004; Pertz et al., 2006; Machacek et al., 2009). Strikingly, as many receptor-based systems rely on secondary messengers that are mutually exclusive, initial symmetry breaking can also occur in the absence of external signaling inputs (Kirschner et al., 2000). One example, how this may be achieved, is the so-called local excitation global inhibition (LEGI) model (Xiong et al., 2010), an extension of the classical reaction-diffusion systems initially described by Alan Turing (Turing, 1952) and later by Meinhardt and Gierer (Gierer and Meinhardt, 1972), where two mutually exclusive states take advantage of minor fluctuations to stochastically induce stable polarized structures.

**FIGURE 1:**
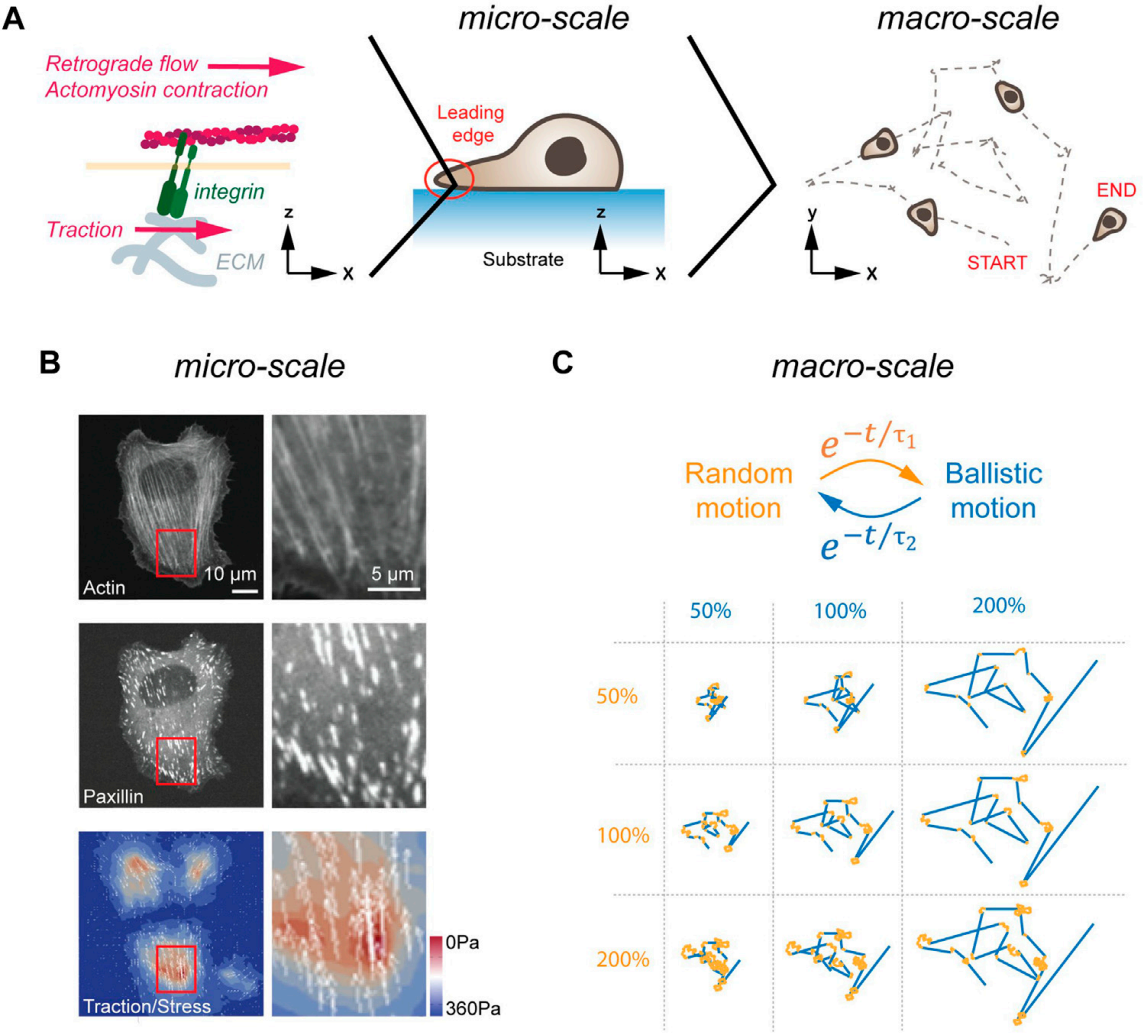
Cell migration in 2D. **(A)** LE dynamics in 2D. From left to right, the molecular clutch, the leading edge of a cell migrating, and a cross-section of a cell migrating on a flat, homogenous surface are shown. The molecular clutch allows to couple the ECM to the cytoskeleton in the presence of tensile forces. The resulting traction is used for cell migration. **(B)** Traction forces of cells plated on elastic substrates. Image of a cell plated on a soft substrate. Image (left) and magnification of a cell stained for actin (top), and the FA protein paxillin (middle), as well as traction forces are shown (bottom). Scale bars, 10 μm (left panels) and 5 μm (right panels). **(C)** Emerging motion pattern at the macro-scale. Cells transition between phases of random (orange) and ballistic (blue) motion. Single trace for different random (orange, duration in percent) and persistent (blue, duration in percent) lifetimes are shown below. Image in **(B)** adapted under CC-BY license from Soiné et al., 2015.

Cell polarization translates into directed cell motion. A result of polarized GTPase activity is a non-isotropic change in actin polymerization dynamics (Peskin et al., 1993) and actin branching (Small et al., 1995), causing cell-wide rearrangements of the cytoskeleton (Figure 1A). Of particular importance for cell motion are changes in actin dynamics at the LE. Considering that the LE has over the last decades been the subject of extensive research, we refer readers interested in this topic to reviews written elsewhere (Pollard and Borisy, 2003; Krause and Gautreau, 2014). Here, we only note that the polymerization rates of individual actin filaments at the LE can reach rates of up to 7 μm/min (Kiuchi et al., 2011), while the LE extends at a rate of only 1–2 μm/min (Begemann et al., 2019). The difference between the polymerization rate of actin and the net forward motion of the LE results in a retrograde actin flow (Lin et al., 1997). This continuous actin treadmilling, further augmented by inward directed actomyosin pulling (Gardel et al., 2008), provides the basic force used for cell propulsion.

Transfer of tractile forces from the cell to the substrate is accomplished by Integrin molecules (Izzard and Lochner, 1976). Integrins are transmembrane receptors, consisting of non-covalently interacting α and *ß* subunits. To date, over 20 unique combinations of α (18 types) and *ß* (8 types) subunits have been described that differ in selectivity to extracellular matrix (ECM) components (Humphries et al., 2006). Common to all Integrins is the ability of participating in bidirectional signaling. Inside-out signaling describes the conformational change in the extracellular domain (i.e., activation) upon binding of Talin to the cytoplasmatic tail of Integrin (Tadokoro et al., 2003). In addition, Integrins also engage in outside-in signaling. Here, Integrins become activated upon force application from outside, which induces conformational changes in Integrin and increased ligand binding affinity (Friedland et al., 2009). In response to this external stimulation, Integrins trigger activation of FAK and other signaling pathways on its cytosolic site (reviewed in Hamidi and Ivaska, 2018). The two central elements for successful force-transduction through Integrins are on the extracellular site the binding of the heterodimer head to the ligand, and on the cytosolic site the unfolding of Talin (del Rio et al., 2009). As individual Integrins display ligand selectivity [e.g., αMβ2 binds ICAM1 (Rosetti et al., 2015); α3β1 binds VCAM1 (Chen et al., 2010)], the former element may give rise to selective cell-ECM interactions. The latter element refers to the fact that Talin only unfolds and binds to Vinculin and to actin in the presence of tensile forces. Hence, only under strain are mechanical forces transmitted from the cytoskeleton to the substrate, a mechanism called the ‘molecular clutch’ (Mitchison and Kirschner, 1988) (Figure 1B). Ultimately, the resulting force asymmetry along the planar cell axis yields forward propulsion, which is further augmented by de-adhesion of focal adhesions (FAs) and retraction at the rear of the cell (Abercrombie, 1980).

Importantly, the LE in most cells is not a stable structure but repeatedly transitions through cycles consisting of extension, adhesion and contraction (Lauffenburger and Horwitz, 1996; Ridley et al., 2003; Stock and Pauli, 2021). With each cycle, the cells crawl only a few micrometers forward. Hence, to efficiently migrate over a long distance in a persistent manner, the LE needs to be continuously reinitiated in the same direction. The need to repeatedly reinitiate the LE is not a design flaw, but a beneficial feature, as periodic extinguishing of activity permits a system to adapt more readily to changing external directional stimuli (Meinhardt and Gierer, 1974; Jilkine and Edelstein-Keshet, 2011). As will be discussed in more detail later, pairing these extension-retraction cycles with differential Integrin activation, caused by environmental factors, can gives rise to distinct changes in LE dynamics.

### 2.2 From force transmission to motion pattern in 2D

The literature presented to this point establishes a mechanism that allows cells to spontaneously polarize and migrate in an arbitrary direction, even in the absence of polarized signaling cues. To properly discuss the relevance of such cell motion, we first need to introduce the concept of self-organization. Following the physico-chemical definition, according to Ilya Prigogine (Nicolis and Prigogine, 1977), self-organization describes the ability of a system to create a spatial or temporal pattern at the macro-scale upon interactions of its components at the micro-scale. Importantly, to qualify for this definition, the emerging properties at the macro-scale need to be fundamentally different than the interactions that drive the pattern formation at the micro-scale, and the system needs to operate far off the thermodynamic equilibrium. Examples that meet these requirements are the animal swarm dynamics (Tunstrøm et al., 2013), and certain patterns arising from reaction-diffusion systems (Turing, 1952). Self-assembling structures, such as crystals, lipid bilayers or polypeptides, which operate near or at the thermodynamic equilibrium, however, do not qualify. Readers interested in learning more about the concept of self-organization, we refer to reviews written elsewhere (Kondo and Miura, 2010; Saha and Galic, 2018; Mancinelli and Galic, 2020).

As summarized above, cells initiate symmetry breaking spontaneously or in response to a polarized input. In the terminology of self-organization, cells employ at the micro-scale cytoskeletal forces at the LE to migrate for a certain amount of time in one direction. At the macro-scale, this causes cells to transition between phases of random and directed motion (Figure 1C). Strikingly, these emerging motion pattern strongly resembles the swimming pattern of *E. coli*, which also transitions stochastically between phases of random and ballistic propulsion (Berg, 1975). As proposed half a century ago, and revisited more recently theoretically, random transitions between phases of persistent and random motion present a search strategy to find sparsely distributed objects in a planar system (Bénichou et al., 2006; Harris et al., 2012). Notably, such a search strategy can also be found in foraging animals (Viswanathan et al., 1996). Here, depending on the distance between individual targets and the detection sensitivity of the searching agent, the lifetime of the persistent phase is adjusted to maximize the search efficiency (Bénichou et al., 2006). Hence, motion patterns arising at the micro-scale from stochastically formed LE can be interpreted at the macro-scale as a search strategy used by cells in pursuit of polarized signaling cues. Importantly, once a chemotactic signal is detected, cellular motion pattern become biased towards the source but maintain a random element (Arrieumerlou and Meyer, 2005), suggesting that stochastic search pattern act as a basic element that is fine-tuned by polarized signaling inputs.

#### 2.2 1 Substrate-dependence of 2D cell dynamics

To this point, we assumed the substrate as uniform with no local or global inhomogeneities, which obviously is an oversimplification, as the environment varies in its adhesiveness, degradability, elasticity, and geometrical properties.

To determine how material properties affect cell dynamics at the micro-sale (Table 1, top left), we take a closer look at force transmission at the LE. As mentioned above, Integrin heads interact selectively with their ligands at the ECM. Importantly, this interaction displays catch-slip-bond dynamics, which means that the lifetime increases with higher tensile forces to an optimum, beyond which it again drops. For example, the maximum lifetime for α5β1 Integrins occurs at 20–30 pN (Kong et al., 2009). Since tensile forces of 5 pN are required to unfold Talin (Yao et al., 2016), the adhesion strength between receptor and ligand is sufficiently strong to trigger the molecular clutch on a rigid surface. On a soft surface, however, deformation of the substrate will preclude Talin unfolding and thus force transmission. Furthermore, assuming a constant pulling force, the absolute tensile force critically relies on the number of Integrin molecules involved in force transmission. Hence, the spatial distribution of ligands will also influence the threshold at which the molecular clutch is engaged. Finally, there are also indirect consequences to be considered. For instance, increased Integrin endocytosis has been observed in response to substrate elasticity, leading to increased rupture of Integrin-ligand complexes on soft substrates (Du et al., 2011).

**TABLE 1:** Modulating cell dynamics at the micro-scale and at the macro-scale. Top panels depicting how changes in 2D substrate properties alter leading edge dynamics (left), as well as some of the corresponding changes in motion pattern (right). Below, changes in cell dynamics at the micro-scale (left) and emerging properties that may arise at the macro-scale (right) are indicated for cells cultured in 3D.

Micro-scale	Macro-scale	
Integrin/ECM: ligand density (Hu et al., 2022)	2D Haptotaxis (Carter, 1965)	2D
Integrin/ECM: stiffness (Schiller et al., 2013)	2D Durotaxis (Lo et al., 2000)	
Integrin/ECM: steric hinderance (Wolf et al. 2013)	2.5D Topotaxis (Park et al., 2016)	
ECM: axial vs transverse deformation	2D Ratchetaxis (Caballero et al., 2015)	
Actin: temperature dependence (Maiuri et al., 2015)	2D Thermotaxis (Khachaturyan et al., 2022)	
Integrin/ECM: ligand density (Zaman et al., 2006)	3D Haptotaxis (Moreno-Arotzena et al., 2015)	3D
Integrin/ECM: stiffness (Zaman et al., 2006)	3D Durotaxis (Raab et al., 2012)	
Integrin/ECM: steric hinderance (Zaman et al., 2006)	3D Topotaxis (Soans et al., 2022)	
ECM: non-isotropic fibers (Fraley et al., 2015)	3D Ratchetaxis (Le Maout et al., 2020)	
Actin: temperature dependence (Maiuri et al., 2015)	3D Thermotaxis (Khachaturyan et al., 2022)	
ECM: plastic vs. elastic deformation (Wisdom et al., 2018)	3D Polarization (Baker and Chen, 2012)	
ECM: pulling at same fiber (Holle et al., 2018)	3D Assembloids (Biggs et al., 2018)	

Differences in material properties alter emerging motion pattern at the macro-scale (Table 1, top right). For instance, depending on the ability of engaging the molecular clutch, cells will move towards areas of higher adhesiveness (haptotaxis; Carter, 1965). Haptotactic behavior may also be induced by changing the density of ligands. Considering that individual Integrins display some degree of selectivity (see above), cells may also respond to spatial changes in ligand-composition of the ECM, even if the global ligand and receptor levels remain constant. In its most extreme case, such changes in ligand levels may lead to 2D cell confinement. Notably, experimental systems that rely on such cell confinement (e.g., micropatterning) present an excellent platform to study systems-level decisions in single and collective cell motion (Segerer et al., 2015; Schreiber et al., 2016; Brückner et al., 2019). Yet, motion patterns are not only regulated through changes in adhesion strength. A second material property that influences the direction towards which cells migrate is the substrate rigidity, a phenomenon called durotaxis (Lo et al., 2000). Depending on the efficiency with which the molecular clutch is engaged, a consequence of ligand binding affinity and Integrin density, cells were reported to display positive (i.e., migration towards higher stiffness) and negative (i.e., migration towards lower stiffness) durotaxis. Importantly, as rigidity is a passive parameter that cannot be directly measured (Elosegui-Artola et al., 2018), cells need to probe the ECM to determine its mechanical properties. This is accomplished using both, lamellipodial as well as filopodia protrusions (Wong et al., 2014). Building on the same theme, cells were further shown to be sensitive to certain topologies (topotaxis; Park et al., 2016). Complementing these mechanisms that rely on variations of the molecular clutch, cells were also described to sense gradients in temperature (thermotaxis; Bahat et al., 2003). While the mechanism remains elusive, it is noteworthy that an increase in temperature yields augmented cell speed and persistence (Maiuri et al., 2015). It is thus plausible that thermotaxis relies on a biased random walk, as observed for chemotaxis.

#### 2.2 2 Substrate-dependence of 3D cell dynamics

As we just elaborated, cells rapidly alter motion dynamics when presented with changing mechanical properties. The same is also true for cells embedded in a 3D environment. Prominent examples are the elevated speed mesenchymal cells display upon being squeezed under a glass slide (Liu et al., 2015) and the increased motility in the presence of a viscous extracellular medium (Bera et al., 2022). To understand the underlying mechanisms, again the material properties of the surrounding substrate need to be considered.

We begin with the similarities at the micro-scale (Table 1, bottom left). One variable that is likely to change is the substrate stiffness, which will drop from a GPa to KPa range when transitioning from rigid glass and plastic dishes to soft gels or tissues (Baker and Chen, 2012). As in a planar system, cells in a 3D environment were shown to move towards higher substrate stiffness (DuChez et al., 2019), and display haptotactic behavior (Moreno-Arotzena et al., 2015). As these parameters appear to be equally important, we will not further pursue them here. Readers interested in learning more on this topic, we refer to excellent reviews written elsewhere (Friedl and Wolf, 2010; Charras and Sahai, 2014; SenGupta et al., 2021; among others). While some parameters remain the same, others change, and a few new ones arise in cells navigating a complex 3-dimensional space. One parameter that changes is the ability of cells to engage with its surroundings on all sides, with potential consequences considering possible asymmetries in global protein distribution. Once cells begin moving through a 3D environment, new parameters need to be faced. For instance, the pore sizes of the ECM through which the cells will squeeze (Wolf et al., 2013) suddenly become relevant. Critical here is the size of the nucleus, as it is the largest organelle of the cell and fragile to mechanical damage. Squeezing the nucleus while moving through a narrow opening not only activates cellular signaling cascades (McGregor et al., 2016), but may in extreme cases yield nuclear rupture causing DNA damage and cell death (Chen et al., 2019). To prevent this, laminin levels that determine the nuclear stiffness were shown to correlate with ECM stiffness (Swift et al., 2013). Conversely, postmitotic and cancer cells that no longer need to protect the nucleus from damage display a soft nucleus (Cross et al., 2007). Where the mesh size is too narrow for cells to move through, secretion of proteases will locally digest the ECM to create space for migration (Gross and Lapiere, 1962). Strikingly, cleavage of collagen through metalloproteases leads to exposure of RGD-containing domains. This, in turn, allows binding to Integrins (Taubenberger et al., 2010) and other ECM molecules (Ortiz Franyuti et al., 2018). Hence, digestion of the environment may not only give space to crawl through, but also reveal adhesion sites needed to generate sufficient traction. Finally, it is noteworthy mentioning that the ECM will also be modified by cells traveling through it. For instance, pulling of cells *via* Integrins on ECM fibers leads to exposure of cryptic binding sites (in fibronectin), which is necessary for its polymerization into fibers (Lemmon et al., 2009). Furthermore, it was shown that aligned ECM fibers are secreted by cells migrating through a 3D environment (Ingman et al., 2006). Intriguingly, since a fiber will more readily give in when pulled perpendicularly, any deviations from a completely isotropic ECM will yield a directional bias, causing ratchetaxis (Caballero et al., 2015). Consequently, the ECM will be subject to modifications as cells travel through it, ultimately changing the trajectories of the cells that will follow, creating a possible memory effect.

Since many mechanisms identified in 2D were also observed in 3D, comparable motion patterns were reported for both systems (Table 1, bottom right). Unfortunately, no systematic analysis of 3D migration patterns exists to date. Considering that mechanical properties within a tissue are not homogenous (i.e., boundary conditions), and as the migrating cell population will inevitably come in contact with resident cell types (i.e., changing mechanical properties, signaling activity), an *ex vivo* approach with precise control of all parameters provides the best strategy to determine how individual constituents affect motion pattern. Yet even an ideal homogenous substrate will show varying pore sizes, obstacles, fiber lengths and stiffnesses, which argues that cell migration in 3D will by default display a larger variance in the motion pattern compared to a 2D system.

#### 2.2 3 Modes of cell migration in 3D

**FIGURE 2:**
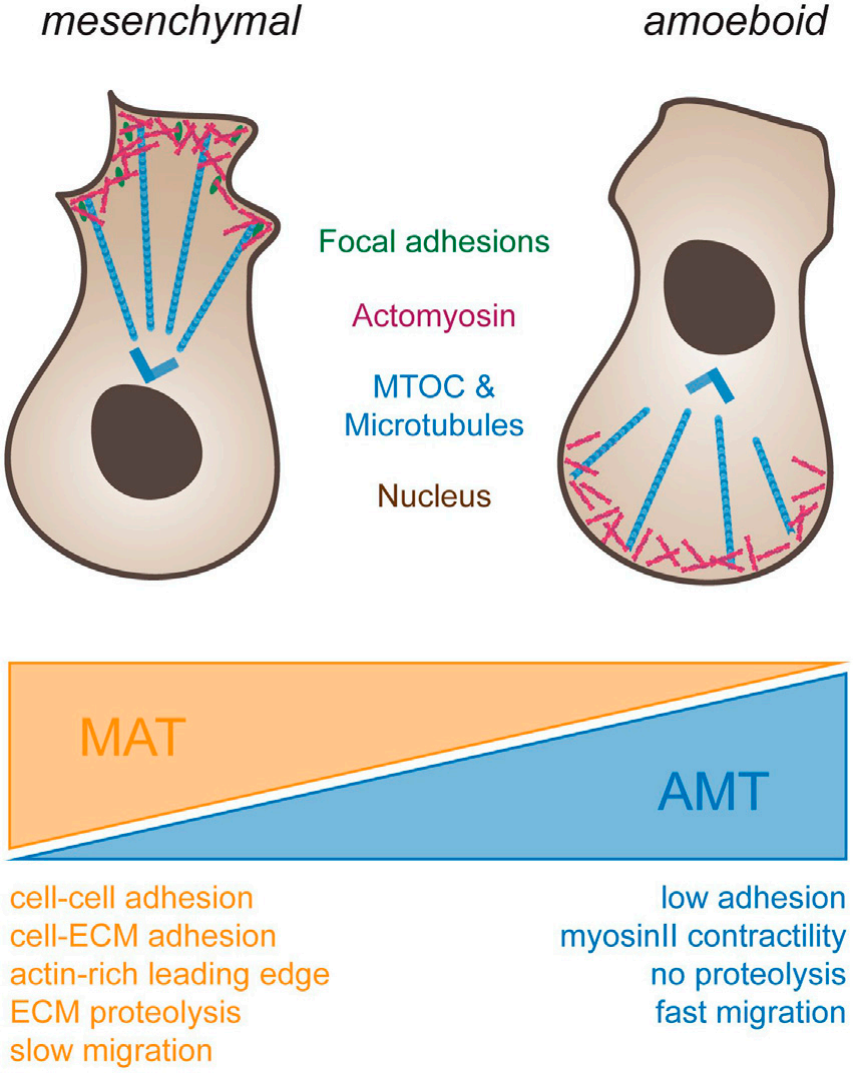
Different types of cell migration. To the left, schematic drawing of a cell using mesenchymal migration. Forces for propulsion are generated by connecting actomyosin-based forces (blue) through focal adhesions (green) to the substrate. Note that the MTOC (blue) is in front of the nucleus. To the right, amoeboid migration is depicted. Cells do not adhere to the substrate but rely on actomyosin-based compression of the cytosol at the back end of the cell for propulsion. Note that in immune cells, such as dendritic cells or macrophages, the MTOC is located behind the nucleus. Transitions from mesenchymal to ameboid migration (orange wedge, mesenchymal-amoeboid transition MAT) are promoted by low adhesion and Rho-ROCK driven Myosin-II activity (Friedl and Wolf, 2010; Graziani et al., 2022). Conversely, transition from amoeboid to mesenchymal migration (blue wedge, amoeboid-mesenchymal transition AMT) are induced by cell-cell and cell-ECM adhesions, actin-rich protrusions at the leading edge driven by Rac and Cdc42, ECM degradability and high Integrin-mediated adhesiveness (Panková et al., 2010; Graziani et al., 2022).

To this point, we were exploring changes in motion from the perspective of a mesenchymal cell. Yet, from a physiological perspective, it is desirable to vary modes of propulsion depending on cell function. For instance, it would be beneficial for immune cells to display low selectivity to the substrate, as they frequently change their local environment on their pursuit of pathogens, while slow moving cells with a specific destination should be more selective. To account for these opposing needs, different types of motion have developed. The most frequent forms are mesenchymal and amoeboid. Mesenchymal cell migration (Figure 2, left), which is observed among others in neural crest cells (Ebendal, 1977) and fibroblasts (Abercrombie, 1980), locates the microtubule organizing center (MTOC) and most organelles in front of the nucleus (Etienne-Manneville and Hall, 2001), and relies as described above on force-transmission through Integrins to the ECM (Doyle et al., 2015).

In contrast, amoeboid cell motion (Figure 2, right), as seen in leukocytes (Norberg et al., 1977) and primordial germ cells (Olguin-Olguin et al., 2021), frequently positions the MTOC and most organelles behind the nucleus (Chiplonkar et al., 1992). Amoeboid migration further relies on short lived adhesions, rather than on mature FA complexes. When inhibiting integrin adhesion to their surroundings, dendritic cells were shown to still migrate in a 3D environment without any change in speed (Lämmermann et al., 2008). However, such cells were impaired in their interaction with endothelial or epithelial surfaces, and displayed reduced resistance to blood flow shear forces, highlighting the importance of Integrins for additional leukocyte-associated functions besides cell migration. When trapped between two planar surfaces, immune cells were further shown to move without FAs by exerting forces on their surroundings, a migration mode termed “chimneying” (Pinner and Sahai, 2009). Here, by applying forces on the surrounding surfaces, sufficient friction is created for traction and locomotion of the cell. In contrast to the mesenchymal migration mode, traction force in this case was generated without the need to couple contraction within the cell to the substrate *via* adhesion complexes. These results point to the existence of subtypes within the amoeboid migration mode, depending on the extent of adhesive, contractile and protrusive forces (Lämmermann and Sixt, 2009). Amoeboid migrating cells make use of protrusive or contractile forces for cell locomotion with varying degrees of adhesiveness, leading to membrane protrusion governed by actin polymerization or by hydrostatic pressure caused by actomyosin contractility (actin-free membrane blebs). Hence, the combination of the degree of adhesion, contraction and protrusion appears to determine the amoeboid migration mode and is cell type specific (Lämmermann and Sixt, 2009).

Intriguingly, the environment does not only affect the mode of migration, but the mode of migration has also a reciprocal effect on the environment (Table 1). Mesenchymal cells are associated with long-lasting remodeling of the ECM. This is due to digestion, as well as due to pulling and rearrangement of the ECM. Amoeboid cells, in contrast, only leave a small imprint as proteolytic activity is minor, and no mechanical forces are exerted on the ECM. Notably, as the ECM is a viscoelastic material, a rapid migration mode may result in an elastic deformation that will close behind the cell, whereas slowly moving mesenchymal cells may yield plastic deformations, which may leave a tunnel, even in the absence of enzymatic modifications.

Importantly, mesenchymal and amoeboid modes should not be considered as mutually exclusive, but rather as two extremes on a continuum. Cells were reported to change between different modes depending on their environment. For instance, some cancer cells switch from mesenchymal to amoeboid upon protease inhibition (Wolf et al., 2003), while others switch from amoeboid to mesenchymal upon increase in the relative HIC5-to-paxillin ratio (Gulvady et al., 2018). Similarly, the viscoelastic properties were reported to change motion dynamics (Petrie et al., 2012; Bera et al., 2022). These findings argue that cells can readily transition between migration modes in response of the level of confinement and adhesion. Consistently, the underlying machinery used for propulsion remains the same. These considerations may also explain the presence of hybrid modes of propulsion such as lobopodial motion (Petrie et al., 2012). Here, similarly to amoeboid migration, cells use hydrostatic pressure to form a blunt spherical protrusion at the cell front, yet rely on pulling forces to the ECM for propulsion. Finally, it is noteworthy to mention the Reynolds number, and its effect on motion at the cellular level. The Reynolds number describes the ratio between the inertia and viscosity of a fluid. Objects at the length scale of cells have a negligible inertia, leaving them to be subject of large viscous forces. Consequently, many cell have adapted to the environment, using non-symmetric motion types to ensure forward movement (Purcell, 1977). Considering this aspect, a more continuous transition between “walking” and “swimming” should be considered, yielding additional forms of propulsion that rely on fluid-like streams (Stroka et al., 2014), or cytoskeletal waves (Barry and Bretscher, 2010). In this review, we only mention these additional modes of motion in passing. Again, we refer readers interested in this topic to excellent reviews written elsewhere (Purcell, 1977; Lauga and Powers, 2009; Caballero et al., 2020).

## 3 Mechanochemical feedback loop in 2D and 3D—A case study

As summarized above, several factors change when transitioning from migration on a planar surface to a three-dimensional environment. To explore how individual parameters may alter cell dynamics, we will take advantage of a curvature-dependent self-organizing circuit, which was previously described to control LE and motion pattern in single mesenchymal cells (Begemann et al., 2019). To properly assess this topic, we first revisit some basic concepts from actin and membrane mechanics.

### 3.1 Curvature-dependent regulation of actin polymerization dynamics

Cell migration in a planar system relies on mechanical forces exerted by the actomyosin network (Gardel et al., 2008). Within the LE, individual actin filaments are facing the cell periphery with their barbed ends (Urban et al., 2010), bringing this heavily regulated end of actin filaments in close proximity to the plasma membrane. This proximity to the site of action provides the system with an intriguing control unit. For one, it allows regulatory elements to be limited to the plasma membrane. More relevant for this review, however, it allows to couple actin dynamics directly to the shape of the membrane itself, thereby bypassing the need for receptor-based signaling cascades.

Cell migration is associated with outward (i.e., negative) and inward (i.e., positive) deformations of the plasma membrane. Such highly curved membrane sections *per se* are energetically not favorable and will rapidly return to their lowest energy state, unless additional circuits are in place to maintain these membrane deformations. Intriguingly, studies identified a protein family consisting of over 70 members that are capable of sensing inward (i.e., positive) and outward (i.e., negative) membrane curvature with the help of a BAR domain (Peter et al., 2004; Bhatia et al., 2009; Galic et al., 2012). Within this protein family, 17 members carry actin-regulatory domains, allowing changes in actin polymerization dynamics in a curvature-dependent manner. Examples regulating actin dynamics at positively curved membrane sites include Arhgap17 and Arhgap44 that accomplish this through its RhoGAP domains (Richnau and Aspenström, 2001; Galic et al., 2014). Complementing these proteins, several I-BAR domain proteins regulate actin polymerization at negatively curved membrane sections through binding of Rho GTPases to the CRIB domain (Zhang et al., 1997), or of actin monomers to the WH2 domain (Marchand et al., 2001). By coupling membrane curvature to actin polymerization dynamics, distinct emerging properties can be achieved. Prominent examples based on stochastic curvature-dependent feedback loops include the emergence of travelling waves (Wu et al., 2018), or the formation of exploratory filopodia in developing neurons (Mancinelli et al., 2021). Intriguingly, recent work established that actin polymerization may depend on the membrane geometry itself. From theoretical (Peskin et al., 1993) and experimental (Bieling et al., 2016) work, we know that individual actin filaments can exert a forced of up to 6 pN. Since forming a protrusion requires more force (Helfrich, 1973; Raucher and Sheetz, 1999), a single filament will only cause shallow membrane deformations. If, however, two (or more) such minor deformations occur in close proximity, membrane tension will cause bundling of individual actin filaments, which may cause the formation of protrusions with multiple actin filaments over time (Liu et al., 2008). In addition, experimental and numerical analysis showed that the shape of the plasma membrane changes the local polymerization rates of branched actin filaments, thus causing the outgrowth of finger-like protrusions (Simon et al., 2019). While limited in number, these experimental and theoretical considerations show the ability of membrane shape to influence actin dynamics and in consequence cell shape and function. Notably, similar self-organization circuits were also described for microtubules (Singh et al., 2018; Gavriljuk et al., 2021).

### 3.2 Curvature-dependent self-organization in 2D

**FIGURE 3:**
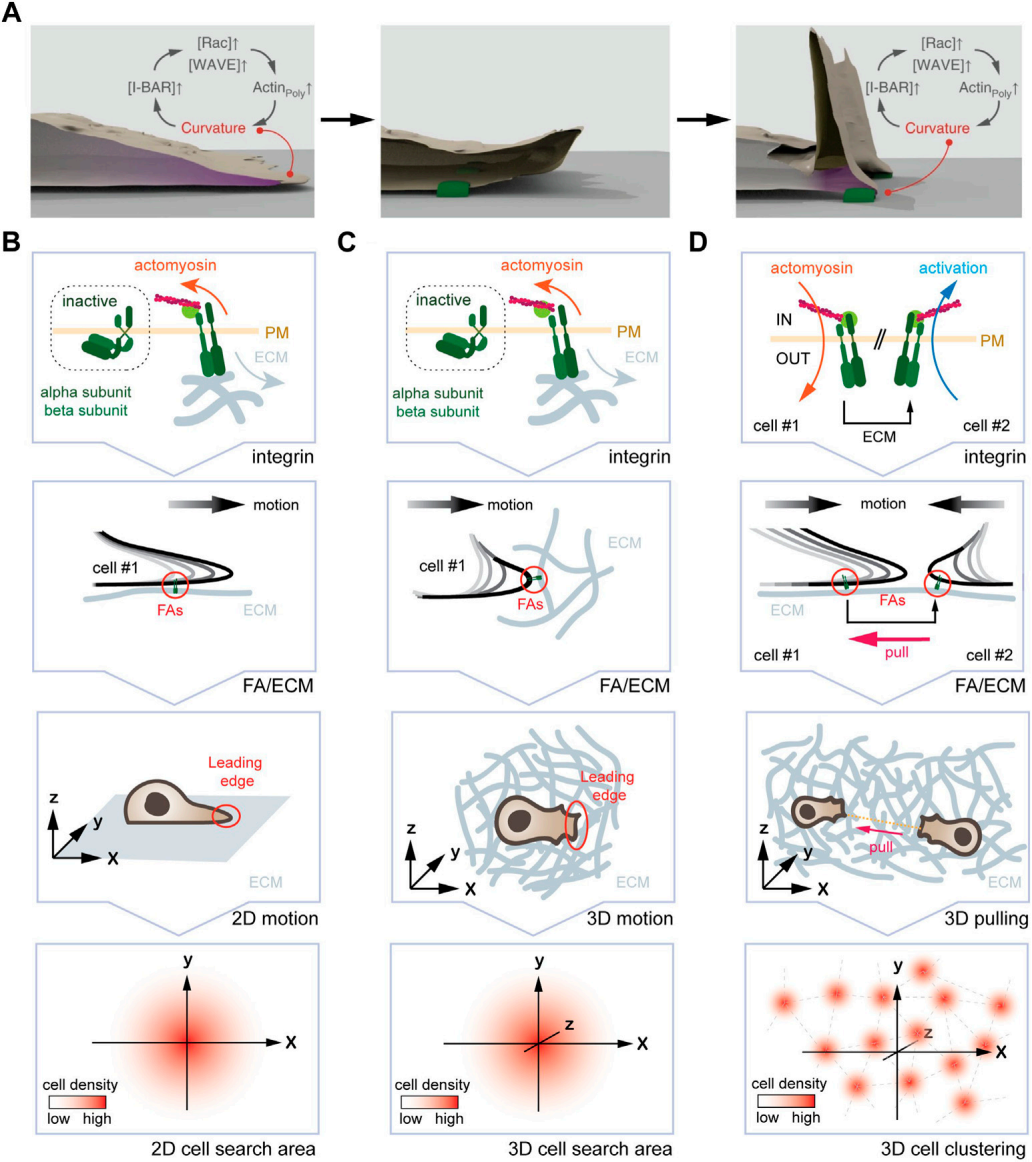
One curvature-dependent self-organizing circuit yields different emerging properties for mesenchymal cells plated in 2D and 3D. **(A)** Self-organizing LE re-initiation circuit in 2D. High local curvature triggers I-BAR domain protein enrichment, which further promotes actin polymerization and protrusion formation. Upon retraction, adhesion of FAs (green) to the ECM leads to a local high plasma membrane curvature, promoting I-BAR protein enrichment, increased actin polymerization, and LE re-initiation. **(B)** From the molecular clutch to motion pattern in 2D. From top to bottom, engagement of the molecular clutch leads to the activation of the curvature-dependent feedback-loop at the LE. The arising motion pattern displays an augmented persistence, which manifests in an increased search efficiency due to lower oversampling of the same areas. **(C)** From the molecular clutch to motion pattern in 3D. From top to bottom, engagement of the molecular clutch, the resulting deformation of the LE, and the arising motion pattern are shown. Again, an increase in persistence time augments the search efficiency. **(D)** Hypothetical mechanism for the formation of cell clusters in 3D. From top to bottom, engagement of the molecular clutch in two adjacent cells is shown. Here, one cell pulling at the ECM will cause inverse membrane deformations in an adjacent cell. This, in turn could activate the self-organizing feedback loop causing cell assembly. Image in **(A)** modified with permission from Begemann et al., 2019.

Keeping in mind the ability of subcellular systems to self-organize in a curvature-dependent manner, we now focus on the LE of migrating cells. Outgrowth of the LE yields outward (i.e., negative) plasma membrane deformations with a curvature of 200–300 nm along the *Z*-axis (Urban et al., 2010). This local negative curvature was reported to trigger assembly of a self-organizing circuit at the tip of the LE (Begemann et al., 2019), which leads to increased actin polymerization and therefore to LE extension (Figure 3A). At the molecular level, this is accomplished through the recruitment of the curvature-sensitive I-BAR domain protein Baiap2 to negative membrane deformation sites. Baiap2 interacts through its CRIB and WH2 domain with actuators of actin polymerization dynamics. Hence, Baiap2 recruitment locally increases actin polymerization dynamics, leading to outgrowth of the LE. While this presents a mechanism for LE extension, it does not explain how the cell terminates this process. One factor, which likely limits the positive feedback loop, is a local increase in membrane tension at the LE (Shi et al., 2018). Published work demonstrates that an increase in membrane tension reduces actin polymerization (Raucher and Sheetz, 2000; Tsujita et al., 2015; Diz-Muñoz et al., 2016). At the same time, increase in membrane tension may also induce mechanically activated ion channels (Coste et al., 2010) to mediate transition from extension to collapsing the LE. Strikingly, the collapse of a retracting LE will cause to the formation of a highly curved membrane site adjacent to adhesion complexes, triggering the enrichment of the signaling machinery and re-initiation of the next LE protrusion. With this mechanochemical feedback loop, the maintenance of the polarization is ensured and therefore the longevity of the LE increased.

This curvature-dependent feedback loop is likely to interface with other regulatory circuits present at the LE. For instance, recent work showed that the retrograde flow of actin filaments at the LE maintains cell polarity, whereby increased polymerization speed (e.g., at higher temperatures) yields faster and more persistent migration (Maiuri et al., 2015). As just mentioned, increased membrane curvature augments recruitment of I-BAR domain proteins, likely resulting in higher actin polymerization rates that will further increase membrane curvature. At the same time, elevated temperatures will reduce membrane rigidity (Lamparter and Galic, 2020), which will lead to an increase in membrane curvature in response to a constant force. It is thus plausible to envision that a curvature-dependent feedback loop may contribute to previously described correlation of actin dynamics and motion persistence of the cell. Yet, published work demonstrates that the retrograde flow does not decrease but increases when Integrin binding is abolished due to actin slippage (Renkawitz and Sixt, 2010), arguing for the presence of additional (compensatory) circuits that work in parallel with the curvature-dependent signaling circuit at the LE.

At the macro-scale, increasing the longevity of LE through repeated re-initiation and other parameters leads to augmented motion persistence (Figure 3B). Augmented persistence, as discussed above, yields increased search efficiency, as it reduces oversampling of the very same position (Begemann et al., 2019).

### 3.3 Curvature-dependent self-organization in 3D

We next discuss how 3D material properties affect this particular self-organizing circuit at the micro- and macro-scale. The LE of mesenchymal cells in 3D resembles structurally the situation in 2D (Thievessen et al., 2015; Doyle et al., 2021). While it remains elusive whether contraction and coordination with subsequent LE extensions occurs in 3D, it is plausible to envision a similar mechanism (Figure 3C). Consistently, the LE of mesenchymal cells grown in a 3D system relies on extension-retraction cycles for forward movement (Doyle et al., 2021). However, LE outgrowth in 3D is not restricted to a plane, but stochastically distributed in all dimensions (Thievessen et al., 2015), thus allowing cells to sample its entire environment for optimal migration conditions (Doyle et al., 2021). Notably, contractile forces are independent of matrix properties (Feld et al., 2020), and single FAs were shown to act as rigidity sensors (Plotnikov et al., 2012). However, while the LE in 2D and 3D both grow out as planar sheets, reduced traction due to lower substrate stiffness is likely to delay and/or reduce membrane curvature at the tip of the LE. Changes in membrane curvature, in turn, may alter protein and isoform composition at the LE. Considering that individual curvature-sensitive proteins bind to distinct target proteins (Safari and Suetsugu, 2012), shape-dependent changes in protein stoichiometry may arise at the LE. Noteworthy, altered protrusion dynamics can be observed in some cell types when transitioning from a stiff 2D to a soft 3D environment (Santos et al., 2020). In the absence of mature FAs, for instance at sites where the ECM surrounding the cell is not suitable to build up sufficient tension, no mesenchymal migration is possible (Thievessen et al., 2015; Doyle et al., 2021). Here, Integrin-independent mechanisms, as shown for a number of cell types (Paluch et al., 2006; Lämmermann et al., 2008), promote cell locomotion. Strikingly, recent work showed that force-induced membrane deformations can lead to Talin-independent activation of integrins (Kim et al., 2020), raising the possibility that Integrins activate upon LE outgrowth to test the strength of the bond by actomyosin contractions. If true, this would leave only the connections that have the optimal conditions for further migration in this direction. For a full understanding of this process, the activation status of Integrins could be imaged during 3D cell migration with focus on the protrusion-adhesion-contraction cycle at the LE.

At the macro-scale, the self-organizing circuit is likely to influence motion pattern as described for the planar system (Begemann et al., 2019). In support of such a notion, published work showed that 3D mesenchymal migration depends on the relative LE adhesiveness (Caballero et al., 2015), whereby FAs will dictate the direction (Lo Vecchio et al., 2020) and the speed (Kim and Wirtz, 2013) of motion. Consistently, ablating collagen fibers 20 µm in front of the LE, which precludes among others re-initiation of the self-organizing circuit, was shown to avoid further migration in this direction (Doyle et al., 2021). How exactly FAs (size, number, maturity, stability, and density) and ECM (composition, density, rigidity, orientation) properties alter LE dynamics and in consequence cell migration, remains elusive. As that the self-organizing circuit is well suited to detect gradients in adhesion strength (haptotaxis), ECM stiffness (durotaxis), and fiber orientation (ratchetaxis), it raises the possibility that it may not only contribute to persistent cell migration but also bias stochastic 3D motion pattern in a particular direction (Figure 3C).

Finally, changing mechanical properties of a 3D system may not only influence motion pattern, but could also yield additional emerging features of the self-organizing circuit. As mentioned above, a cell pulling on the substrate will deform the adjacent matrix (Doyle et al., 2021). For two neighboring cells that adhere to the same fiber, this means that one cell will sense the other cell tugging the fiber (Figure 3D). As pulling the fiber away from a cell will form an outward membrane deformation, this may trigger activation of the self-organizing circuit and LE extension in that direction. Hence, it is plausible to envision that pulling on the same fiber may yield self-organization of cells into equidistant cell aggregates, which has been observed *in vitro* (Trappmann et al., 2017) and *in vivo* (Biggs et al., 2018; Trela et al., 2021). To determine this putative emerging property, the activation status of Integrins in neighboring cells could be imaged, and corresponding motion pattern determined.

## 4 Concluding remarks

In this review we surveyed the changing mechanical properties encountered by cells when transitioning from a 2D to a 3D environment and discussed its functional consequences. While incomplete, the presented data demonstrates that the ECM-cell interactions are by no means linear. For instance, increasing the concentration of an ECM component in a hydrogel will not only change the material properties (i.e., rigidity, mesh size), but also the ligand density, and may also induce receptor endocytosis. Furthermore, reducing the mesh size beyond a certain threshold may lead to increased protease secretion, which would not only soften the ECM but also expose new ligands for Integrin-binding. Considering this intricate interdependence of material and cellular parameters, the ECM-cell system should be considered a N-dimensional space with numerous distinct constellations that cells populate (and transition in-between) to efficiently navigate the 3D space.

How to untangle such an interdependent system? Considering its importance at the microscale, a promising first step towards a comprehensive understanding of 3D cell migration is a better grasp on Integrin-ligand dynamics. Towards this goal, microscopic studies and biophysical measurements of biomimetic hydrogels will be essential to clearly separate individual parameters influencing 3D cell migration, and to determine the hierarchy of these factors. Such quantitative measurements will further be crucial for developing future numerical models that recapitulate an idealized 3D environment–a critical prerequisite towards exploring optimal search strategies in three dimensions. Such a quantitative approach, however, hinges on the development of suitable 3D microscopy techniques, as well as an extensive description of the matrix properties. Only such a systematic analysis will provide us with a holistic view of motion patterns in 3D, and may on the long turn open up new inroads to study cell dynamics under physiological (e.g., immune system; Tabdanov et al., 2021) and pathological (e.g., cancer metastasis; Eddy et al., 2021) conditions.
